# Short-Term Efficacy and Tolerability of Paroxetine Versus Placebo for Panic Disorder: A Meta-Analysis of Randomized Controlled Trials

**DOI:** 10.3389/fphar.2020.00275

**Published:** 2020-03-31

**Authors:** Beilin Zhang, Chao Wang, Lexiang Cui, Jiguo Gao, Chenglin Wang, Xiangyu Tan, Shaokuan Fang

**Affiliations:** Department of Neurology, Neuroscience Centre, The First Teaching Hospital of Jilin University, Changchun, China

**Keywords:** efficacy, tolerability, paroxetine, panic disorder, meta-analysis

## Abstract

**Objective:**

To explore the short-term efficacy and tolerability of paroxetine in the treatment of panic disorder in adults.

**Methods:**

Multiple electronic databases were searched to find randomized controlled trials (RCTs) on paroxetine and panic disorder. The primary efficacy outcomes were: the mean change compared to the baseline in the total number of full panic attacks, Clinical Global Impression-Severity of Illness (CGI-S) score, and the proportion of participants with zero full panic attacks and with a 50% or greater reduction in the number of full panic attacks. The tolerability outcomes included withdrawal rate and the incidence of adverse events (AEs).

**Results:**

13RCTs were included. The pooled analyses showed patients who received paroxetine experienced greater improvements in the number of full panic attacks (total: MD=-1.96, 95%CI -3.45 to -0.47, P=0.010; ≥50% reduction: OR=1.66, 95%CI 1.08 to 2.55, P=0.02; zero full panic attacks: OR=1.70, 95%CI 1.42 to 2.03, P < 0.00001) and CGI-S (MD=-0.37, 95%CI -0.74 to -0.01, P=0.05) than placebo. There was no evident difference in the total withdrawal rate (OR=0.91, 95%CI 0.76 to 1.08, P=0.26) and withdrawal rate due to AEs (OR=1.29, 95%CI 0.97 to 1.72, P=0.07) between the two groups. Withdrawal rate due to lack of efficacy or relapse (OR=0.44, 95%CI 0.31 to 0.63, P < 0.00001) and the incidence of serious AEs (OR=0.42, 95%CI 0.23 to 0.79, P=0.007) in the paroxetine group was lower than the placebo group. Meanwhile, the incidence of any treatment-emergent adverse events (TEAEs) (OR=1.32, 95%CI 1.05 to 1.64, P=0.02) in the paroxetine group was higher in comparison with the placebo.

**Conclusions:**

Paroxetine is an effective and well-tolerated short-term treatment for adults with panic disorder.

## Introduction

Panic disorder (PD) is a common mental health disorder with a lifetime prevalence of 1.6% to 2.2% in the general population (American Psychiatric Association, 2009), while in primary care settings, panic syndromes have been reported to have a prevalence of around 10% (King et al., 2008). PD is characterized by repeated, unexpected panic attacks, which are discrete periods of fear or anxiety that have a rapid onset, reach a peak within 10 minutes, and in which at least 4 of 13 characteristic symptoms are experienced (Bighelli et al., 2018). Many of these symptoms involve bodily systems, such as a racing heart, chest pain, sweating, shaking, dizziness, flushing, stomach churning, faintness, and breathlessness (Bighelli et al., 2018). Unless the clinician has a high index of suspicion, panic disorder may remain undetected (Tsuboi and Masuko, 2001). PD is also reported to be associated with comorbid major depression disorder and its prevalence is 24%-88%, which will increase the risk of suicidal behavior (Dannon et al., 2004).

Selective serotonin reuptake inhibitors (SSRIs) are a known first-line therapy for PD. Among them, paroxetine was first approved by the U.S Food and Drug Administration for the treatment of PD. It can selectively inhibit the re-uptake of serotonin in the presynaptic membrane, and thus increase the concentration of serotonin in the synaptic gap and enhance the central 5-hydroxytryptaminergic neurological function. To date, a series of randomized, double-blind, placebo-controlled clinical trials have been carried out to study its short-term efficacy and tolerability, however there are still relatively few quantitative analyses on it. Therefore, we conducted a meta-analysis and systematically reviewed the relevant literature to evaluate the short-term efficacy and tolerability of paroxetine in order to provide evidence for clinical treatment and research of PD in adults.

## Methods

The following work was conducted according to the preferred reporting items for systematic reviews and meta-analyses (PRISMA) statement (Moher et al., 2009). The PROSPERO registration number is: CRD42019145115.

### Criteria for Considering Studies for This Review

The inclusion criteria were as follows: (1) participants had to be patients aged 18 years or older with a principal diagnosis of PD, with or without agoraphobia, and met any of the following criteria: International Classification of Disease-10 (ICD-10), or Diagnostic and Statistical Manual of Mental Disorders (DSM-III,DSM-IIIR,DSM-IV); (2) an intervention in which paroxetine was used as monotherapy with a duration of less than 6 months. There was no restriction on dose and frequency; (3) a placebo was used as a comparison; (4) the outcome measures were indicators of efficacy and tolerability. The article might be included in the meta-analysis as long as it had one of the above data. (5) randomized controlled trials (RCTs) were used as the study types.

Studies were excluded if they included patients with: (1) severe physical disease; (2) another axis I disorder instead of panic disorder; (3) recent major depression disorder (unless panic attacks were deemed to be the predominant disorder and preceded affective symptoms chronologically); (4) Hamilton Depression(HAMD) scale(17-item) total score≥18; (5) drug or alcohol dependence or abuse within the last 6 months; (6) substantial suicide risk; (7) use of anxiolytics and/or other antidepressants within 2 weeks before the randomized treatment; (8) poor response or hypersensitivity to a SSRI in the past; (9) receiving paroxetine or electroconvulsive therapy 3 months before study entry; or cognitive behavioral therapy within 30 days before randomization. Studies from which we could not obtain enough valid information were also removed.

### Search Methods for Identification of Studies

#### Electronic Searches

These electronic databases were searched from their inception to July 25, 2019: PubMed, Web of Science, Embase, Cochrane Central Register of Controlled Trials, ScienceDirect, Scopus, PsycINFO, Wanfang, China Biology Medicine disc (CBMdisc), Chongqing VIP, and China National Knowledge Infrastructure (CNKI). We limited the languages to Chinese and English, without any restrictions on publication status. The search terms were: (paroxetine OR seroxat OR cylert OR BRL29060 OR FG-7051 OR brisdelle OR LDMP OR pexeva OR paxil OR aropax) AND (“panic disorder” OR “panic attack” OR agoraphobia OR “acute anxiety”). Medical Subject Headings (MESH) words and free words were used to improve recall ratio. We also searched Clinical Trials.gov to find unpublished studies. Details of the search strategy were shown in **Supplementary S1 file** and the same search strategy was used to retrieve the above databases again on August 31 to discover if there were any new literature that needed to be included. We have translated the search terms of Chinese databases into corresponding English to show it better.

#### Searching Other Resources

The references of the relevant systematic reviews, meta-analyses, and all the included literature were also searched in order to avoid missing any possibly necessary literature.

### Data Collection and Analysis

#### Selection of Studies

Two authors used End Note X9 and manually removed any duplicate literature independently. Then we browsed the titles and abstracts of remaining documents to exclude literature that did not conform to inclusion criteria. At last, we read the full text of the remaining literature after screening carefully to determine the final included literature and clarify the specific reasons for the removal. If there was any disagreement during this process, it was necessary to reach a consensus after a consultation with the third member.

#### Data Extraction and Management

These data were summarized in an Excel spreadsheet: the first authors, publication year, study design, diagnostic criteria, location, intention-to-treat and completed numbers, gender and age of participants, intervention and control measures, treatment duration, and indicators of efficacy and tolerability. The above work was carried out by two individuals independently and a third person helped them negotiate and resolve any disputes that may have arisen.

#### Assessment of Risk of Bias in Included Studies

The Cochrane Collaboration’s tool for assessing risk of bias in randomised trials (Higgins et al., 2011) consists of six domains: selection bias (random sequence generation, allocation concealment), performance bias (blinding of participants and personnel), detection bias (blinding of outcome assessment), attrition bias (incomplete outcome data), reporting bias(selective reporting), and other biases. Two members independently evaluated each domain with “low risk”, “high risk”, or “unclear risk”. If they could not come to an agreement, the decision should be made after discussion with the third member. “High risk of bias” may alter the results seriously, “low risk of bias”, if present, is unlikely to alter the results seriously, and “unclear risk of bias” means it would raise some doubt about the results (Higgins et al., 2011).

#### Measures of Treatment Effect

We focused on the efficacy and tolerability of paroxetine compared with a placebo in the treatment of PD. The primary efficacy was mainly reflected by the mean change compared to the baseline in the total number of full panic attacks and Clinical Global Impression-Severity of Illness (CGI-S) scale score, as well as the proportion of participants with zero full panic attacks and with a 50% or greater reduction in the number of full panic attacks. Secondary efficacy outcomes included mean change compared to the baseline of various scales, the intensity of anticipatory anxiety, as well as response and remission rate. The response rate was defined as a Clinical Global Impression-Improvement (CGI-I) rating of “very much improved” or “much improved” and the remission rate was defined as CGI-S rating of “not at all ill” or “borderline ill” and no Panic and Anticipatory Anxiety Scale (PAAS) full-symptom panic attacks (Pollack et al., 2007b). Tolerability was defined as the withdrawal rate and the incidence of AEs.

Dichotomous data: the subjects with zero full panic attacks, the proportion of participants with a 50% or greater reduction in the number of full panic attacks, response and remission rate, total withdrawal rate, withdrawal rate due to a lack of efficacy or relapse, withdrawal rate due to AEs, the incidence of any treatment-emergent adverse events (TEAEs), serious adverse events (SAEs), and common TEAEs.

Continuous data: mean change compared to the baseline for the following data: total number of full panic attacks, intensity of anticipatory anxiety, Hamilton Anxiety (HAMA) Scale total score, Sheehan Disability Scale (SDS) work, social life and family life scores, CGI-S score, CGI-I score, Marks Sheehan Phobia Scale(MSPS)overall phobia, total avoidance and fear scores, Montgomery-Asberg Depression Rating Scale (MADRS) score, and Social Adjustment self-report Questionnaire (SAQ) score.

#### Unit of Analysis Issues

The studies we included were all randomized parallel-group trials.

If a trial involved multiple treatment groups, such as three or more arms trial that investigated different dose of paroxetine and placebo, or compared two different antidepressants and placebo, we would only compare the paroxetine arm with placebo or each dose of paroxetine with placebo, respectively.

#### Dealing With Missing Data

For dichotomous variables, if the original literature did not provide the specific number of events, but provided the events rate, we would calculate the number of events for analysis. For continuous variables, if the literature only provided the standard error (SE) rather than the standard deviation (SD), we would calculate the SD according to the following formula: $\mathrm{SE}=SD/\sqrt{n}$. (n is the corresponding sample size in each data).

If there were still some missing data that affected our analysis in the included literature, we would contact the original authors to obtain the corresponding information.

#### Assessment of Heterogeneity

We used chi-square (Chi^2^) test and its P value as well as I-square (I^2^) value to evaluate heterogeneity. I^2^≥0% is non-heterogeneous, I^2^≥25% is considered to be mild heterogeneity, I^2^≥50% is considered to be moderate heterogeneity, and I^2^≥75% is considered to be severe heterogeneity (Shi et al., 2019). P=0.10 is used as a threshold of statistical significance (Bighelli et al., 2018).

#### Assessment of Reporting Biases

Reporting biases arise when the dissemination of research findings is influenced by the nature and direction of results (Sterne et al., 2008). Our meta-analysis mainly involved the following two types of reporting biases: publication bias and outcome reporting bias. The former is the publication or non-publication of research findings and the latter is selective reporting of some outcomes instead of others, both depending on the nature and direction of the outcomes (Sterne et al., 2008).

Funnel plots were used to visually reflect the presence or absence of publication bias; however, it should not be used when the number of studies was less than 10. Begg’s and Egger’s test were used for quantitative assessment of publication bias. P < 0.05 indicated the publication bias was significant (Shi et al., 2019).

To detect selective outcome reporting bias, we compared the outcomes of published literature with their trial registries, if they were available. Otherwise, we compared the methods and outcomes in the publications. If all prespecified outcomes were reported, we could consider it as “low risk”.

#### Data Synthesis

The Review Manager (RevMan) 5.3 software was used in our data synthesis. Dichotomous data were pooled by the Mantel-Haenszel (M-H) statistical method and odds ratio (OR) with a 95% confidence interval (CI). Continuous data were processed by the Inverse Variance statistical method and mean difference (MD) with 95% CI. The fixed-effect model was applied by default and the random-effects model was used in case of substantial heterogeneity (I^2^≥50% and P < 0.10) (Hugues et al., 2017). The overall effect was calculated by Z test, thereof P value ≤ 0.05 means statistically significant.

#### Subgroup Analysis and Investigation of Heterogeneity

Subgroup analysis is a common method to deal with heterogeneity. If the pooled data suggested significant heterogeneity, we need to look for sources of heterogeneity. In our meta-analysis, if there were enough studies available, we could conduct subgroup analysis based on the dose or duration of paroxetine.

#### Sensitivity Analysis

To assess the robustness of our outcomes and to explore the contribution of each included trial, sensitivity analysis was conducted by omitting the studies one by one and recalculating the data using Stata version 15.1 (Tabrizi et al., 2019).

## Results

### Description of Studies

#### Results of the Search

**Figure 1:**
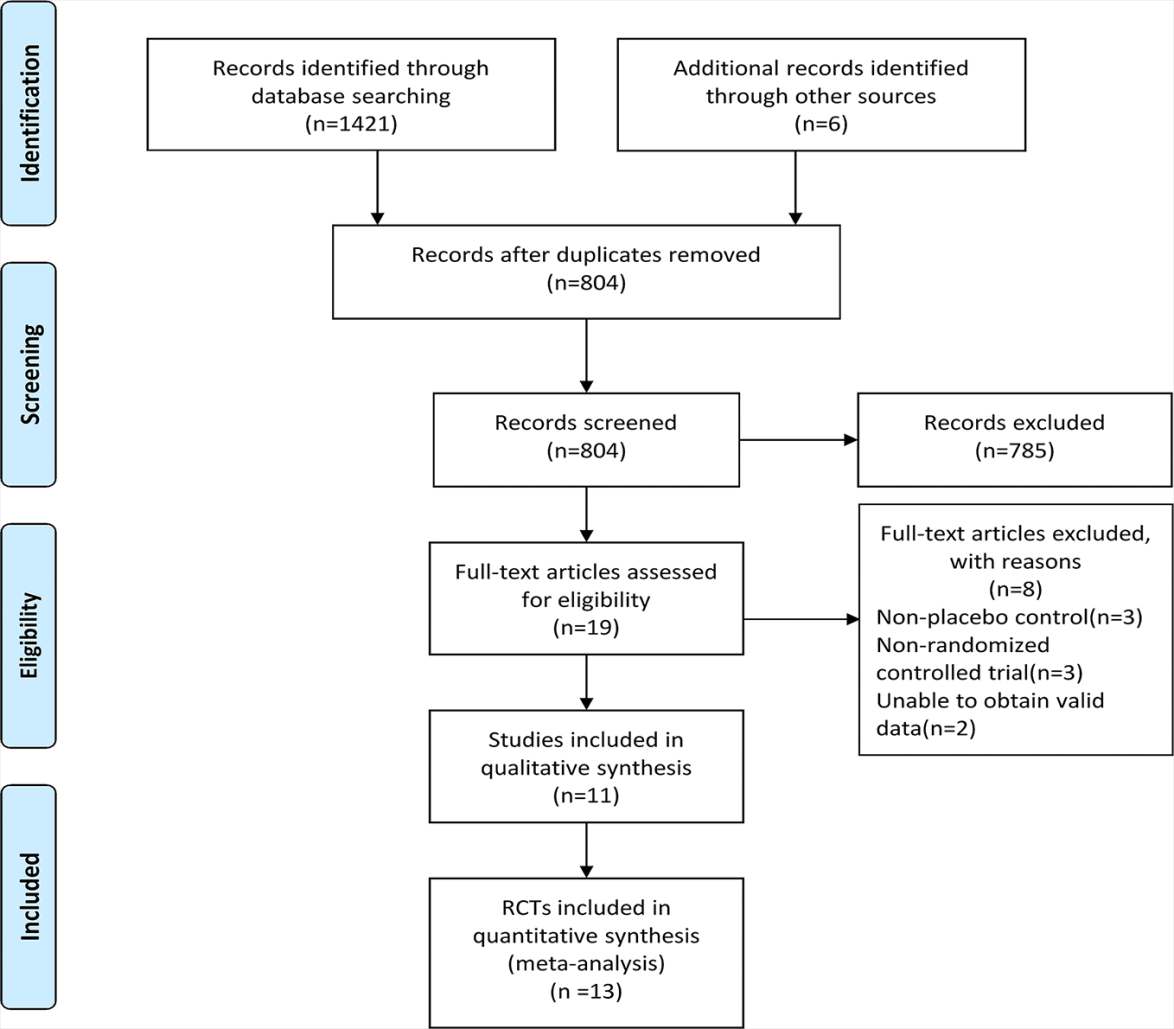
PRISMA flow diagram.

A total of 1421 records were identified through the electronic database searching and 6 additional documents were found after retrieving the reference list of relevant systematic review, meta-analyses, and included studies. After removing the duplicates by using End Note X9 software and manually, 804 records remained. We next browsed the titles and abstracts of records left and 785 records that did not meet the inclusion criteria were removed.We also read the full-text of 19 articles to evaluate their eligibility, then 8 articles were excluded and 11 studies were included for qualitative synthesis. 13 RCTs were finally included in the meta-analysis. See **Figure 1** for PRISMA flow diagram.

#### Included Studies

All included studies were multicenter, randomized, double-blind, parallel-group placebo-controlled trials. They were conducted from 1994 to 2007. The total number of intention-to-treat (ITT) population in 13 RCTs were 2654, among which 1329 were in the intervention group and 1325 were in the control group. Mean age ranged from 34.7 to 45.0 years. 4 studies were three arms, 4 studies were four arms, and 3 studies were two arms. The fixed dose of paroxetine was in 6 RCTs and flexible dose in 7 RCTs. The maximum dosage reached 75mg. Course of treatment ranged from 9 weeks to 12 weeks. Details of the included RCTs were shown in **Table 1**.

**Table 1:** The characteristics of included RCTs in the meta-analysis.

Study	Case (I/C)	Gender (M/F)	Age (I/C) (years)	Diagnostic criteria	Intervention/control	Paroxetine dose (mg/d)	Durance (weeks)	Outcome	Multicenter	Study design
Lecrubier et al. (1997)	123/123	102/144	34.7 ± 9.3/35.0 ± 8.9	DSM-III-R	paroxetine/placebo	20-60	12	(1)(6)(8)(9)(10)(14)(15)(16)(17)(18)	Y	RCT
Pollack et al. (2007a)	151/157	97/211	37.5 ± 11/35.1 ± 9.48	DSM-IV	paroxetine/placebo	40	12	(4)(12)(13)(14)(15)(16)(17)(18)	Y	RCT
Pollack et al. (2007b)	161/156	106/211	37.6 ± 10.5/37.7 ± 11.3	DSM-IV	paroxetine/placebo	40	12	(4)(12)(13)(14)(15)(16)	Y	RCT
Ballenger et al. (1998)	67/69	42/94	36.1 ± 9.1/37.3 ± 10.4	DSM-III-R	paroxetine/placebo	10	10	(1)(2)(3)(4)(5)(6)(7)(8)(9)(10)(11)(12)(14)(15)(16)(17)(18)	Y	RCT
Ballenger et al. (1998)	70/69	46/93	35.9 ± 10.1/37.3 ± 10.4	DSM-III-R	paroxetine/placebo	20	10	(1)(2)(3)(4)(5)(6)(7)(8)(9)(10)(11)(12)(14)(15)(16)(17)(18)	Y	RCT
Ballenger et al. (1998)	72/69	51/90	36.3 ± 10.8/37.3 ± 10.4	DSM-III-R	paroxetine/placebo	40	10	(1)(2)(3)(4)(5)(6)(7)(8)(9)(10)(11)(12)(14)(15)(16)(17)(18)	Y	RCT
Judge and Dunbar (1995)	123/123	NA	NA	DSM-III-R	paroxetine/placebo	10-60	12	(14)(17)	Y	RCT
Bakker et al. (1999)	32/32	21/43	34.7 ± 8.9/35.1 ± 7.6	DSM-III-R	paroxetine/placebo	20-60	12	(1)(2)(6)(8)(10)(14)(15)	Y	RCT
Sheehan et al. (2005a)	158/163	113/208	36.51 ± 10.09/36.60 ± 10.68	DSM-IV	Paroxetine CR/placebo	12.5-75	10	(4)(6)(8)(12)(14)(15)(16)(17)(18)	Y	RCT
Sheehan et al. (2005b)	147/138	121/164	38.2± 10.4/40.1 ± 10.7	DSM-IV	Paroxetine CR/placebo	12.5-75	10	(4)(6)(8)(12)(14)(15)(16)(17)(18)	Y	RCT
Sheehan et al. (2005c)	139/144	122/161	38.1 ± 10.0/37.0 ± 10.0	DSM-IV	Paroxetine CR/placebo	12.5-75	10	(4)(6)(8)(12)(14)(15)(16)(17)(18)	Y	RCT
GlaxoSmithKline (1994)	77/72	51/98	39.1 ± 11.1/39.0 ± 11.8	DSM-III-R	paroxetine/placebo	10-60	10	(1)(2)(3)(4)(5)(6)(7)(8)(9)(10)(11)(14)(15)(16)(17)(18)	Y	RCT
Bergink and Westenberg (2005)	9/10	8/11	44/45	DSM-IV	paroxetine HCL/placebo	60	9	(4)(14)(15)(16)	Y	RCT

RCTs, randomized controlled trials; I/C, intervention/control; M, male; F, female; DSM, diagnostic and statistical manual of mental disorders; Y, yes; CR, controlled release; HCL, hydrochloride; (1) total number of full panic attacks; (2) Clinical Global Impression-Severity of illness (CGI-S) total score; (3) the proportion of patients with a 50% or greater reduction in the number of full panic attacks; (4) the proportion of patients with zero full panic attacks; (5) the intensity of anticipatory anxiety; (6) Hamilton Anxiety Scale (HAMA) total score; (7) Clinical Global Impression-Improvement (CGI-I) total score; (8) Marks Sheehan Phobia Scale (MSPS) scores; (9) Sheehan Disability Scale (SDS) score; (10) Montgomery-Asberg Depression Rating Scale (MADRS) score; (11) Social Adjustment self-report Questionnaire (SAQ) total score; (12) response rate; (13) remission rate; (14) total withdrawal rate; (15) withdrawal rate due to lack of efficacy or relapse; (16) withdrawal rate due to adverse events; (17) the incidence of any treatment-emergent adverse events; (18) the incidence of serious adverse events.

#### Excluded Studies

8 articles were excluded after reading the full-text for the following reasons: non-placebo control arm (n=3), non-randomized controlled trial (n=3), unable to obtain valid data (n=2).

### Risk of Bias in Included Studies

**Figure 2:**
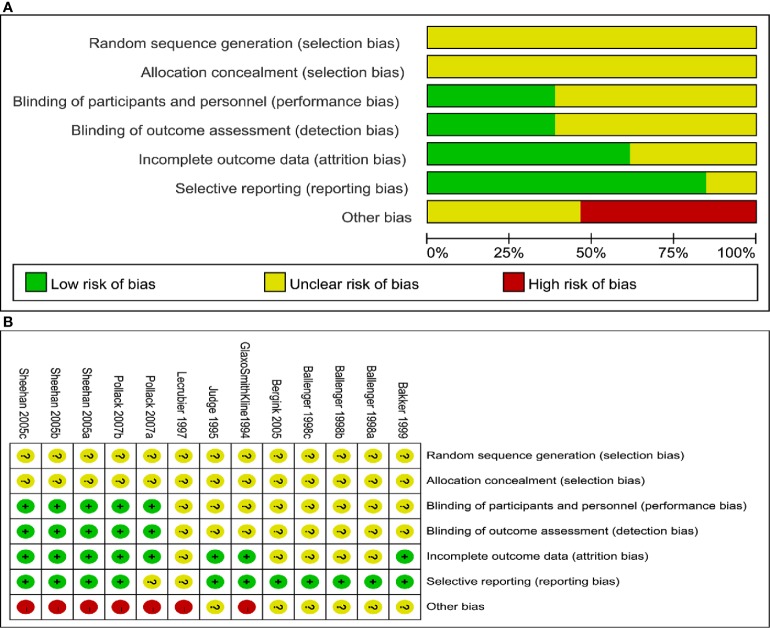
**(A)** Risk of bias graph: review authors’ judgements about each risk of bias item presented as percentages across all included studies, **(B)** Risk of bias summary: review authors’ judgements about each risk of bias item for each included study.

The outcomes of the risk of bias assessment were presented in two forms: risk of bias graph (**Figure 2A**) and risk of bias summary (**Figure 2B**).

### Effects of Interventions

#### Primary Efficacy Outcomes

**Figure 3:**
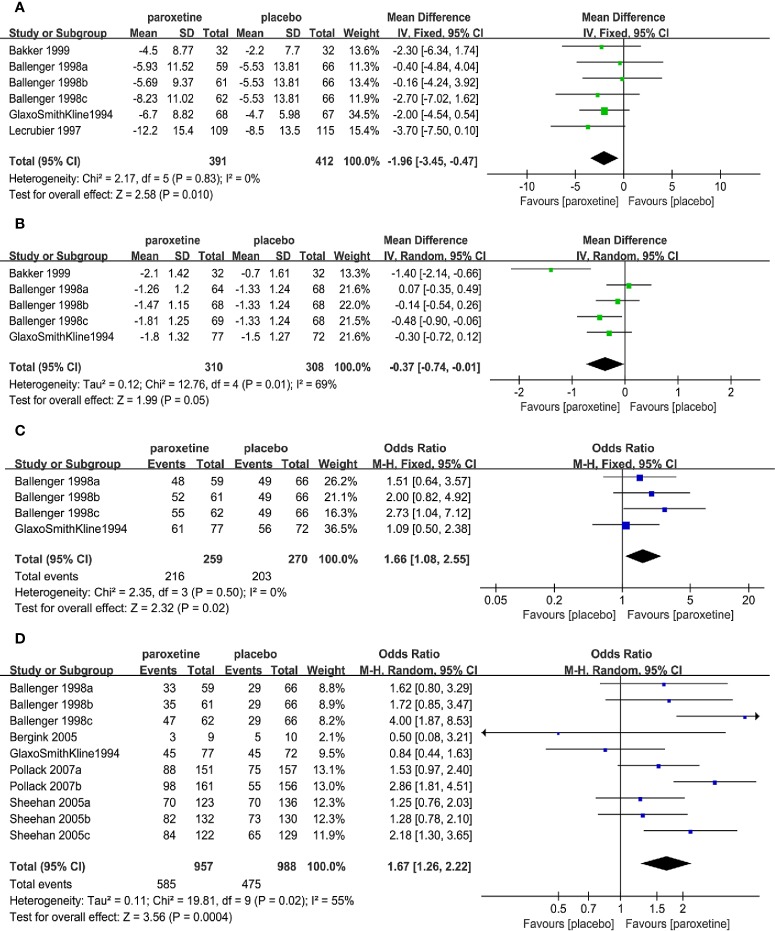
Forest plot of primary efficacy outcomes. **(A)** mean change from baseline in total number of full panic attacks, **(B)** mean change from baseline in CGI-S total score, **(C)** the proportion of patients with a 50% or greater reduction in the number of full panic attacks, **(D)** the proportion of patients with zero full panic attacks.

Mean change compared to the baseline in the total number of full panic attacks (6RCTs, MD=-1.96, 95%CI -3.45 to -0.47, P=0.010) (**Figure 3A**) and CGI-S total score (5RCTs, MD=-0.37, 95%CI -0.74 to -0.01, P=0.05) (**Figure 3B**)in the paroxetine group was higher in comparison with the placebo group. No heterogeneity was detected in the former (P=0.83, I^2^ = 0%), while the heterogeneity was evident in the latter (P=0.01, I^2^ = 69%), therefore we changed to the random-effects model. There was no statistically significant difference of their baseline between the two groups (former: P=0.77; latter: P=0.68).

The proportion of patients with a 50% or greater reduction in the number of full panic attacks (4RCTs, OR=1.66, 95%CI 1.08 to 2.55, P=0.02) (**Figure 3C**) and the subjects with zero full panic attacks (10RCTs, OR=1.67, 95%CI 1.26 to 2.22, P=0.0004) (**Figure 3D**) in the paroxetine group was higher in comparison with the placebo group. The former had no heterogeneity (P=0.50, I^2^ = 0%), while the latter had substantial heterogeneity (P=0.02, I^2^ = 55%), therefore the random-effects model was applied.

#### Secondary Efficacy Outcomes

**Figure 4:**
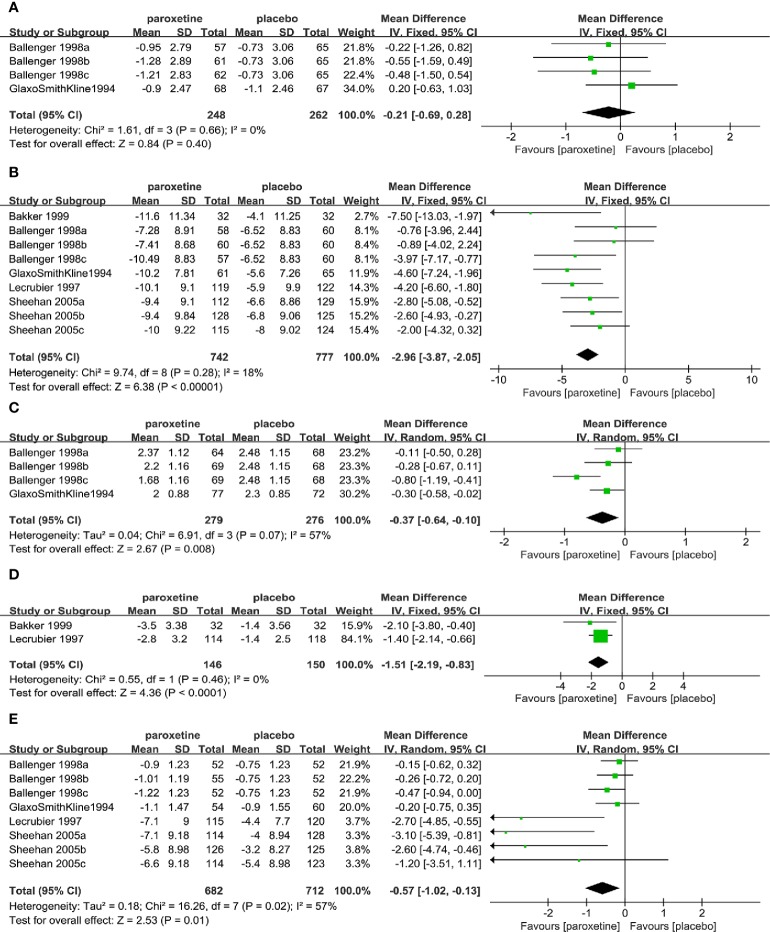
Forest plot of secondary efficacy outcomes.**(A)**the intensity of anticipatory anxiety, **(B)** HAMA total score, **(C)** CGI-I total score, **(D)** MSPS overall phobia, **(E)** MSPS total avoidance scores.

There was no significant difference in the mean change compared to the baseline in intensity of anticipatory anxiety (4RCTs, MD=-0.21, 95%CI -0.69 to -0.28, P=0.40) between the two groups (**Figure 4A**). No heterogeneity was detected (P=0.66, I^2^ = 0%). There was also no statistically significant difference of its baseline between the two groups (P=0.92).

Mean change compared to the baseline of HAMA total score (9RCTs, MD=-2.96, 95%CI -3.87 to -2.05, P < 0.00001) (**Figure 4B**) and CGI-I total score (4RCTs, MD=-0.37, 95%CI -0.64 to -0.10, P=0.008) (**Figure 4C**) in the paroxetine group was higher than that in placebo group. No substantial heterogeneity was identified in the former (P=0.28, I^2^ = 18%). For the latter, on account of its significant heterogeneity (P=0.07, I^2^ = 57%), we applied the random-effects model. There was no statistically significant difference of former baseline between the two groups (P=0.21).

Mean change compared to the baseline of MSPS overall phobia (2RCTs, MD=-1.51, 95%CI -2.19 to -0.83, P < 0.0001) (**Figure 4D**) and total avoidance (8RCTs, MD=-0.57, 95%CI -1.02 to -0.13, P=0.01) (**Figure 4E**) scores in the paroxetine group were higher than that in the placebo group. No heterogeneity was identified in the overall phobia item (P=0.46, I^2^ = 0%). However, another item had substantial heterogeneity (P=0.02, I^2^ = 57%), therefore we used the random-effects model. There was no statistical difference in baseline values between the two items (avoidance: P=0.49; overall phobia: P=0.70). Otherwise, the baseline values provided by the fear item were statistically different (P=0.007), therefore we didn’t make any further comparisons on it.

**Figure 5:**
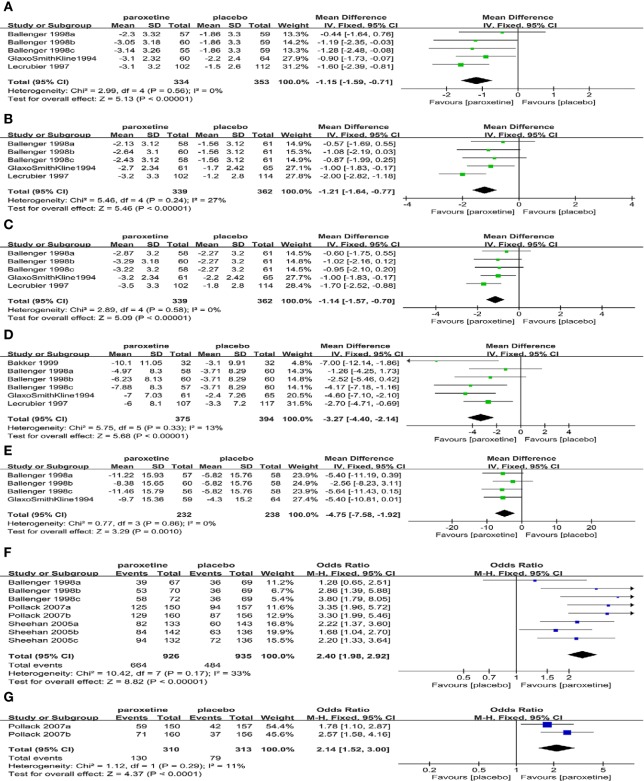
Forest plot of secondary efficacy outcomes. **(A)** SDS work scores, **(B)** SDS family life score, **(C)** SDS social life score, **(D)** MADRS score, **(E)** SAQ total score, **(F)** response rate, **(G)** remission rate.

Mean change compared to the baseline of SDS work (5RCTs, MD=-1.15, 95%CI -1.59 to -0.71], P < 0.00001) (**Figure 5A**), family life (5RCTs, MD=-1.21, 95%CI -1.64 to -0.77], P < 0.00001) (**Figure 5B**), and social life (5RCTs, MD=-1.14, 95%CI -1.57 to -0.70], P < 0.00001) (**Figure 5C**)scores in the paroxetine group were greater than that in the placebo group. No substantial heterogeneity was found in the three items (work: P=0.56, I^2^ = 0%; family life: P=0.24, I^2^ = 27%; social life: P=0.58, I^2^ = 0%). There was no statistical difference in baseline values among the three domains (work: P=0.13; family life: P=0.45; social life: P=0.58).

Mean change compared to the baseline of MADRS total score (6RCTs, MD=-3.27, 95%CI -4.40 to -2.14], P < 0.00001) (**Figure 5D**), and SAQ total score (4RCTs, MD=-4.75, 95%CI -7.58 to -1.92, P=0.0010) (**Figure 5E**) in the paroxetine group was higher in comparison with the placebo group. No heterogeneity was identified (MADRS: P=0.33, I^2^ = 13%; SAQ: P=0.86, I^2^ = 0%). There was no statistically significant difference in baseline values between the two groups (MADRS: P=0.17; SAQ: P=0.80).

The response (8RCTs, OR=2.40, 95%CI 1.98 to 2.92], P < 0.00001) (**Figure 5F**) and remission rate (2RCTs, OR=2.14, 95%CI 1.52 to 3.00, P < 0.0001) (**Figure 5G**) in the paroxetine group were better than in the placebo group. No substantial heterogeneity was identified (response: P=0.17, I^2^ = 33%; remission: P=0.29, I^2^ = 11%).

#### The Tolerability Outcomes

##### Withdrawal Rate

**Figure 6:**
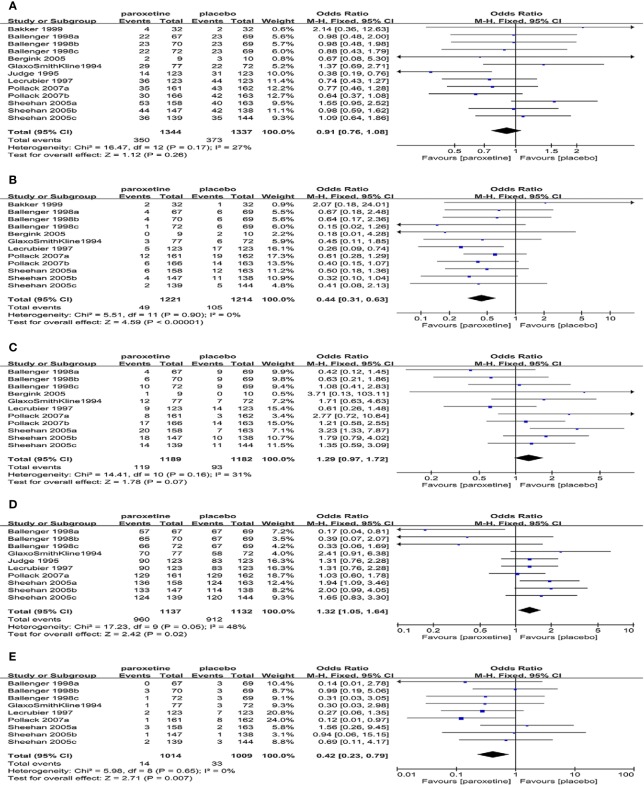
Forest plot of tolerability outcomes. **(A)** total withdrawal rate, **(B)** withdrawal rate due to lack of efficacy or relapse, **(C)** withdrawal rate due to AEs, **(D)** the incidence of any TEAEs, **(E)** the incidence of SAEs.

All RCTs described total withdrawal rate, and the results showed that the total withdrawal rate between the two groups was not statistically significant (13RCTs, OR=0.91, 95%CI 0.76 to 1.08, P=0.26) and no substantial heterogeneity was detected (P=0.17, I^2^ = 27%) (**Figure 6A**).The withdrawal rate due to a lack of efficacy or relapse was reported in 12 RCTs, while no heterogeneity was detected after a pooled analysis(P=0.90, I^2^ = 0%), and the results showed that fewer patients in the paroxetine group were withdrawn due to a lack of efficacy or relapse than those in the placebo group, which was statistically significant (12RCTs, OR=0.44, 95%CI 0.31 to 0.63, P < 0.00001) (**Figure 6B**). There was no obvious difference in withdrawal rates due to AEs between the two groups (11RCTs, OR=1.29, 95%CI 0.97 to 1.72, P=0.07), and we didn’t find any substantial heterogeneity (P=0.16, I^2^ = 31%) (**Figure 6C**).

##### The Incidence of AEs

The incidence of any TEAEs in the paroxetine group was higher than that in the placebo group (10RCTs, OR=1.32, 95%CI 1.05 to 1.64, P=0.02) (**Figure 6D**). No substantial heterogeneity (P=0.05, I^2^ = 48%) was found.

The incidence of SAEs in the paroxetine group was lower than that in the placebo group (9RCTs, OR=0.42, 95%CI 0.23 to 0.79, P=0.007) (**Figure 6E**) and no heterogeneity was detected (P=0.65, I^2^ = 0%).

Common TEAEs reported in the included RCTs were analyzed. There was no significant difference in sinusitis, constipation, respiratory disorder, decreased appetite, nervousness, headache, and dizziness between the two groups. The incidence of dysmenorrhea, infection, and dyspepsia in the paroxetine group was lower than that in the placebo group. Finally, the incidence of sweating, nausea, dry mouth, somnolence, insomnia, diarrhea, female genital disorders, decreased libido, erectile dysfunction, asthenia, tremor, and abnormal ejaculation in the paroxetine group was higher than that in the placebo group. In the pooled analyses of sinusitis and constipation, we used the random effects model due to the heterogeneity detected. See **Table 2** for details. We listed the common AEs associated with the use of paroxetine in **Table 3** based on the rank of incidence.

**Table 2:** Meta-analyses of common TEAEs reported in the included RCTs.

TEAEs	RCTs (N)	OR [95%CI]	Heterogeneity	Effect model	Overall effect (P value)
Female genital disorders^*^	3	11.07[2.03-60.46]	P = 0.99 I^2^ = 0%	Fixed	P = 0.006
Dysmenorrhea^*^	3	0.32[0.11-0.91]	P = 0.61 I^2^ = 0%	Fixed	P = 0.03
Dyspepsia	3	0.22[0.10-0.50]	P = 0.33 I^2^ = 11%	Fixed	P = 0.0003
Decreased libido	2	2.37[1.14-4.93]	P = 0.22 I^2^ = 32%	Fixed	P = 0.02
Infection	4	0.57[0.34-0.96]	P = 0.12 I^2^ = 49%	Fixed	P = 0.03
Decreased appetite	5	1.42[0.84-2.38]	P=0.78 I^2^ = 0%	Fixed	P = 0.19
Respiratory Disorder	7	0.83[0.60-1.14]	P = 0.92 I^2^ = 0%	Fixed	P = 0.25
Nervousness	6	0.9[0.62-1.31]	P = 0.36 I^2^ = 9%	Fixed	P = 0.58
Sinusitis	3	1.11[0.30-4.05]	P = 0.08 I^2^ = 60%	Random	P = 0.88
Erectile dysfunction^*^	2	8.85[1.10-71.49]	P = 0.63 I*^2^* = 0%	Fixed	P = 0.04
Abnormal ejaculation^*^	8	11.5[5.70-23.20]	P = 0.96 I^2^ = 0%	Fixed	P < 0.00001
Diarrhea	9	1.74[1.27-2.38]	P = 0.14 I^2^ = 35%	Fixed	P = 0.0005
Somnolence	9	2.11[1.63-2.74]	P = 0.32 I^2^ = 14%	Fixed	P < 0.00001
Insomnia	8	1.64[1.26-2.13]	P = 0.79 I^2^ = 0%	Fixed	P = 0.0002
Asthenia	8	1.93[1.41-2.65]	P = 0.61 I^2^ = 0%	Fixed	P < 0.0001
Tremor	6	3.86[2.12-7.05]	P = 0.89 I^2^ = 0%	Fixed	P < 0.0001
Constipation	7	1.47[0.73-2.98]	P = 0.02 I^2^ = 60%	Random	P = 0.28
Dizziness	9	1.05[0.78-1.41]	P = 0.27 I^2^ = 19%	Fixed	P = 0.75
Headache	8	0.85[0.68-1.06]	P = 0.12 I^2^ = 39%	Fixed	P = 0.14
Sweating	3	2.79[1.67-4.67]	P = 0.19 I^2^ = 41%	Fixed	P < 0.0001
Nausea	9	1.3[1.03-1.64]	P = 0.73 I^2^ = 0%	Fixed	P = 0.03
Dry mouth	9	1.71[1.29-2.26]	P = 0.29 I^2^ = 17%	Fixed	P = 0.0002

TEAEs, treatment-emergent adverse events; RCTs, randomized controlled trials; OR, odds ratio; CI, confidence interval; ^*^Corrected for gender.

**Table 3:** Common AEs associated with paroxetine.

Common AEs	Number	Proportion (%)
Nausea	200	16.99
Somnolence	196	16.65
Insomnia	167	14.19
Dry mouth	147	12.49
Asthenia	121	10.28
Diarrhea	114	9.69
Abnormal ejaculation	76	6.46
Sweating	57	4.84
Tremor	52	4.42
Decreased libido	26	2.21
Female genital disorders	13	1.10
Erectile dysfunction	8	0.68
Total	1177	100

AE, adverse events.

### Publication Bias

**Figure 7:**
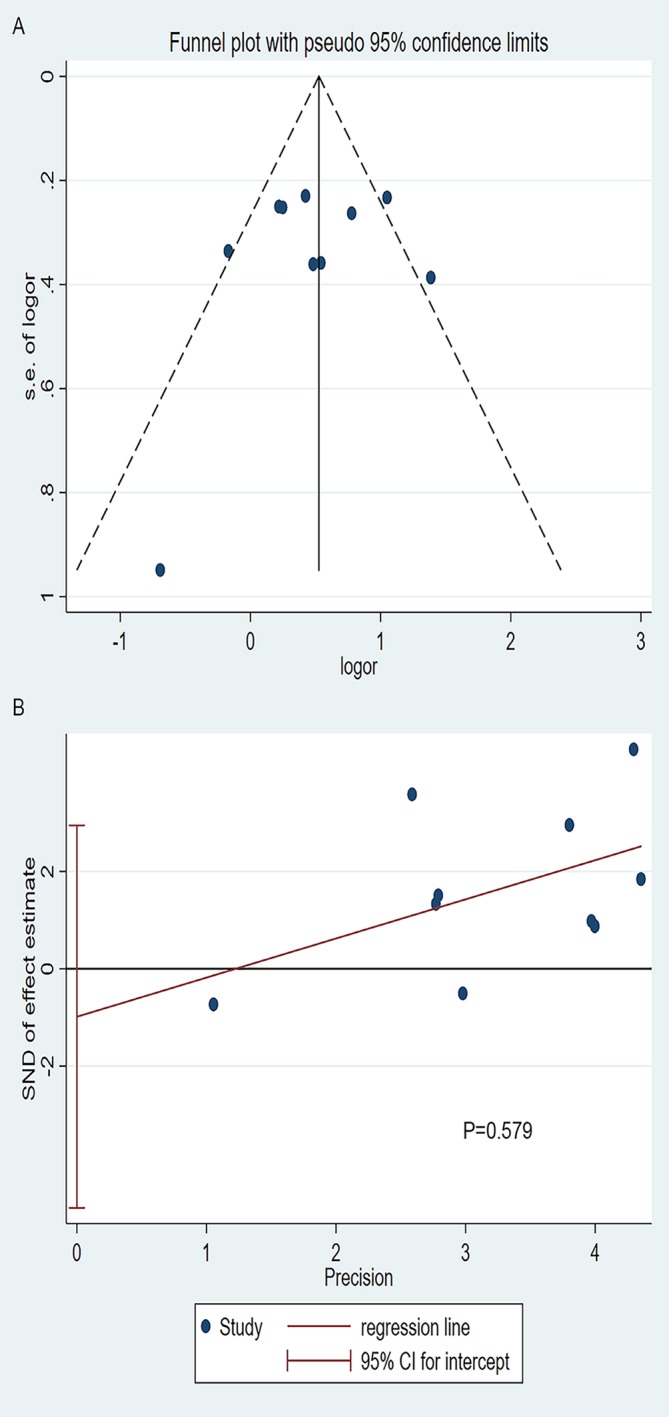
**(A)** The funnel plot of publication bias, **(B)** Egger’s test of publication bias.

The visual examination of the funnel plot (**Figure 7A**) suggested that there might be publication bias and the cause was mainly the existence of small sample studies. But the Begg’s and Egger’s test (**Figure 7B**) did not support the result (Begg’s: P=1.000, Egger’s: P=0.579).

### Sensitivity Analyses

We conducted sensitivity analyses for all the results with substantial heterogeneity, and the results were stable (see **Supplementary S2 file**).

## Discussion

### Summary of Main Results

Through the quantitative analyses of 13RCTs, we found patients who received paroxetine experienced more significant improvement in the frequency of full panic attacks, HAMA, MADRS, SAQ, MSPS, overall phobia and avoidance, CGI-S, CGI-I, SDS work, social life, and family life scores, as well as response and remission rate than those who received the placebo. There was no evident difference in the intensity of anticipatory anxiety, total withdrawal rate, and withdrawal rate due to AEs between the two groups. In addition, withdrawal rate due to a lack of efficacy or relapse and the incidence of SAEs in the paroxetine group was lower in comparison with the placebo group. Meanwhile, the incidence of any TEAEs in the paroxetine group was higher in comparison to the placebo group. The most common TEAEs were sweating, nausea, diarrhea, dry mouth, somnolence, female genital disorders, decreased libido, insomnia, asthenia, impotence, tremor, and abnormal ejaculation. Other TEAEs such as sinusitis, constipation, infection, respiratory disorder, dizziness, dyspepsia, headache, dysmenorrhea, decreased appetite, and nervousness were not related to paroxetine.

### Overall Completeness and Applicability of Evidence

We searched eligible trials as comprehensively as possible, especially unpublished trials. All the included 11 RCTs reported the pre-set results completely and provided detailed data which we could analyze, besides the remaining 2 RCTs which were finally considered as “unclear”. In short, none of the RCTs were identified as high risk of bias in terms of selective outcome reporting. Therefore, we could conduct pooled analyses of multiple items on enough trials to prove that paroxetine is effective and well tolerated in the short-term therapy of adults with PD, mainly reflected in the frequency of panic attacks, anxiety, depression, social functions, withdrawal rate, AEs, and so on.

In our research, 2654 participants entered the final analysis, and the sample size was large enough that the conclusions could be basically extended to most other patients with similar conditions. Our conclusions were also consistent with the guidelines, and paroxetine is a commonly used clinical medication for adults with panic disorder. In terms of tolerability, we found that patients treated with paroxetine had a lower incidence of SAEs than placebo, although the incidence of any TEAEs was higher than placebo, and there was no marked difference in the withdrawal rate due to AEs between the two groups. Hence, paroxetine was regarded as effective and well- tolerated and as something that could be applicable to clinical practice.

### Quality of the Evidence

All the studies that we included were multicenter, randomized, double-blind, placebo-controlled trials, thus the quality of evidence was relatively high in theory, although a lot of research did not provide a detailed scheme of random allocation and blinding, so therefore the overall quality was mixed and unclear. It’s still worth noting that the main high risk of bias was in other bias domain. Furthermore, the authors stated the studies were funded by pharmaceutical companies, and so consequently there might be a certain degree of overestimation of efficacy. Overall, none of the trials had a significant methodological bias, thus the quality of evidence could be considered good.

### Potential Biases in the Review Process

There were some limitations that need to be noted. Firstly, we did not stratify the factors that might cause heterogeneity, such as the dosage and dosage form of paroxetine, although we did sensitivity analysis on items with substantial heterogeneity, and the results were stable. Secondly, we only included English and Chinese studies, so we might have missed out high-quality studies in other languages. Finally, as for publication bias, the results of the visual examination of the funnel plot and Begg’s as well as Egger’s test were contradictory, although we have searched all eligible published and unpublished studies as thoroughly as possible, we could not rule out that some studies with small samples, negative results, or sponsored by pharmaceutical companies have not been detected.

### Agreements and Disagreements With Other Studies or Reviews

To our knowledge, this was the first meta-analysis of paroxetine versus placebo directly and individually in the treatment of PD, although paroxetine had been included in previous systematic reviews and meta-analyses. Our results were generally consistent with previous studies (Andrisano et al., 2013; Sugarman et al., 2014; Bighelli et al., 2018), however we made a more comprehensive comparison between paroxetine and placebo in terms of improving panic symptoms, anxiety, depression, and social function and other aspects. It’s worth mentioning that the methodological quality of Andrisano’s research might not be very high, because they included both non-randomized and non-placebo controlled studies (Andrisano et al., 2013; Bighelli et al., 2018). In addition, there was a contradiction between our meta-analysis and Andrisano’s research regarding the total withdrawal rate, while their results after the sensitivity analysis was so consistent with ours that there was no evident difference between the two groups, which to some extent supported the credibility of our results.

## Conclusions

### Implications for Practice

Based on the available data, we could conclude that paroxetine was effective and well-tolerated in the short-term treatment of adults with PD. We believe that the evidence we provided will be of some benefit to clinical practice. However, the maximum duration of treatment of studies included in this meta-analysis lasted only 12 weeks and it did not include data on long-term treatment because that might increase heterogeneity, while paroxetine usually lasts for a long time in clinical application, so our results cannot be directly applied to the evaluation of efficacy and tolerability for paroxetine in long-term treatment. Besides, it is important for us to comprehensively consider the actual condition of patients when we choose the drugs due to individual differences in each patient.

### Implications for Research

This meta-analysis only compared paroxetine as a monotherapy with placebo, however there are still many clinical options on treating PD, so head-to-head comparisons of multiple medications for PD (or combined with psychotherapy) and network meta-analyses will be necessary to determine the order of their efficacy and tolerability in the future. Further refinement and stratification of the dose for paroxetine is also required to make recommendations for an optimal therapeutic dose.

## Author Contributions

BZ, CW, and LC contributed to the conception and design of the study. JG and CLW organized the database. LC and XT performed the statistical analysis. BZ wrote the first draft of the manuscript. CW wrote sections of the manuscript. All authors contributed to manuscript revision, read, and approved the submitted version.

## Funding

This work was supported by the National Natural Science Foundation of China (81873794).

## Conflict of Interest

The authors declare that the research was conducted in the absence of any commercial or financial relationships that could be construed as a potential conﬂict of interest.
