# Supplementary material for: Short-Term Efficacy and Tolerability of Paroxetine Versus Placebo for Panic Disorder: A Meta-Analysis of Randomized Controlled Trials
Source: Front Pharmacol. 2020 Mar 31;11:275. doi: 10.3389/fphar.2020.00275 (PMC7136560; doi:10.3389/fphar.2020.00275)
Supplement: Supplementary file 1 [file DataSheet_1.docx]

**Supplementary S1 file.** Detailed Search Strategy

Search strategy in PubMed

#1 paroxetine [Mesh]——3913

#2 (seroxat OR FG-7051 OR paxil OR aropax OR BRL-29060 OR "BRL 29060" OR "FG 7051 " OR "paroxetine acetate" OR "paroxetine hydrochloride anhydrous" OR "paroxetine maleate" OR "paroxetine, cis-(+)-Isomer" OR "paroxetine, cis-(-)-Isomer" OR "paroxetine, trans-(+)-Isomer" OR "paroxetine hydrochloride hemihydrate" OR "paroxetine hydrochloride, hemihydrate" OR "paroxetine hydrochloride") [Title/Abstract]——170

#3 #1 OR #2——3968

#4 "panic disorder/drug therapy " [Mesh]——1285

#5 ("panic attack" OR "disorder, panic" OR "disorders, panic" OR "panic disorders" OR "panic attacks" OR "attack, panic" OR "attacks, panic") [Title/Abstract]——4042

#6 "agoraphobia/drug therapy"[Mesh]——456

#7 agoraphobias [Title/Abstract]——6

#8 #4 OR #5 OR #6 OR #7——5060

#9 (((((((("Randomized Controlled Trial"[Publication Type]) OR "controlled clinical trial"[Publication Type]) OR randomized[Title/Abstract]) OR "Clinical Trials as Topic"[Mesh:NoExp]) OR randomly[Title/Abstract]) OR trial[Title]) OR placebo[Title/Abstract]))——1241024

#10 #3 AND #8 AND #9——63

Search strategy in Web of Science

#1 (TS=(paroxetine OR seroxat OR paxil OR brisdelle OR pexeva OR FG-7051 OR LDMP OR aropax OR 61869-08-7 OR BRL-29060 OR BRL29060 OR "BRL 29060" OR "FG 7051" OR FG7051 OR "paroxetine acetate" OR "paroxetine hydrochloride anhydrous" OR "paroxetine maleate" OR "paroxetine, cis-(+)-Isomer" OR "paroxetine, cis-(-)-Isomer" OR "paroxetine, trans-(+)-Isomer" OR "paroxetine hydrochloride hemihydrate" OR "paroxetine hydrochloride, hemihydrate" OR "paroxetine hydrochloride" OR paxpar)) AND publication type:(clinical trial) AND language: (English OR Chinese)——1604

#2 (TS=("panic disorder" OR "panic attack" OR "disorder, panic" OR "disorders, panic" OR "panic disorders" OR "panic attacks" OR "attack, panic" OR "attacks, panic" OR "agoraphobia" OR "acute anxiety" OR panic OR fear)) AND publication type:(clinical trial) AND language: (English OR Chinese)——6578

#3 #1 AND #2——166

Search strategy in Embase

('panic'/exp OR 'panic':ti,ab OR 'panic attack':ti,ab OR 'panic disorder':ti,ab OR 'agoraphobia'/exp OR 'agoraphobia':ti,ab OR 'agoraphobic anxiety':ti,ab OR 'agoraphobic fear':ti,ab OR 'phobia, agora':ti,ab) AND (ldmp:ti,ab OR 'paroxetine'/exp OR '4 (4 fluorophenyl) 3 [ (3, 4 methylenedioxyphenoxy) methyl] piperidine':ti,ab OR 'arketis':ti,ab OR 'aropax':ti,ab OR 'aropax 20':ti,ab OR 'aroxat':ti,ab OR 'brisdelle':ti,ab OR 'brl 29060':ti,ab OR 'brl 29060a':ti,ab OR 'brl29060':ti,ab OR 'brl29060a':ti,ab OR 'daparox':ti,ab OR 'deroxat':ti,ab OR 'dexorat':ti,ab OR 'divarius':ti,ab OR 'dropax':ti,ab OR 'euplix':ti,ab OR 'eutimil':ti,ab OR 'fg 7051':ti,ab OR 'fg7051':ti,ab OR 'frosinor':ti,ab OR 'motivan':ti,ab OR 'optipar':ti,ab OR 'paluxetil':ti,ab OR 'paluxon':ti,ab OR 'paroc':ti,ab OR 'parogen':ti,ab OR 'paroxedura':ti,ab OR 'paroxet':ti,ab OR 'paroxetin':ti,ab OR 'paroxetina':ti,ab OR 'paroxetine':ti,ab OR 'paroxetine hydrochloride':ti,ab OR 'paroxetine mesilate':ti,ab OR 'paroxetine mesylate':ti,ab OR 'paroxia (drug)':ti,ab OR 'paxan':ti,ab OR 'paxil':ti,ab OR 'paxil cr':ti,ab OR 'paxtine':ti,ab OR 'paxxet':ti,ab OR 'pexeva':ti,ab OR 'sereupin':ti,ab OR 'seroxat':ti,ab OR 'setine':ti,ab OR 'si 211103':ti,ab OR 'si211103':ti,ab OR 'solben (drug)':ti,ab OR 'syntopar':ti,ab OR 'tagonis':ti,ab) AND ('placebo'/exp OR 'placebo':ti,ab OR 'placebos':ti,ab) AND ('randomized controlled trial'/de OR 'controlled trial, randomized' OR 'randomised controlled study' OR 'randomised controlled trial' OR 'randomized controlled study' OR 'randomized controlled trial' OR 'trial, randomized controlled' OR 'controlled clinical trial'/de OR 'clinical trial, controlled' OR 'controlled clinical comparison' OR 'controlled clinical drug trial' OR 'controlled clinical experiment' OR 'controlled clinical study' OR 'controlled clinical test' OR 'controlled clinical trial' OR 'clinical trial (topic)'/de OR 'clinical trial (topic)' OR 'clinical trials' OR 'clinical trials as topic' OR 'multicenter study'/de OR 'multi-center study' OR 'multi-center trial' OR 'multi-centre study' OR 'multi-centre trial' OR 'multicenter study' OR 'multicenter trial' OR 'multicentre study' OR 'multicentre trial' OR 'study, multicenter' OR 'trial, multicenter' OR 'controlled study'/de OR 'control group study' OR 'control group trial' OR 'controlled study' OR 'controlled trial' OR 'controlled clinical trial (topic)'/de OR 'controlled clinical trial (topic)' OR 'controlled clinical trials' OR 'controlled clinical trials as topic' OR 'randomized controlled trial (topic)'/de OR 'randomized controlled trial (topic)' OR 'randomized controlled trials' OR 'randomized controlled trials as topic') AND ([chinese]/lim OR [english]/lim) AND [embase]/lim——162

Search strategy in Cochrane Central Register of Controlled Trials

#1 paroxetine [Mesh]——932

#2 (seroxat OR cylert OR FG-7051 OR brisdelle OR LDMP OR pexeva OR paxil OR aropax OR BRL-29060 OR BRL29060 OR "BRL 29060" OR "FG 7051" OR FG7051 OR "paroxetine acetate" OR "paroxetine hydrochloride anhydrous" OR "paroxetine maleate" OR "paroxetine, cis-(+)-Isomer" OR "paroxetine, cis-(-)-Isomer" OR "paroxetine, trans-(+)-Isomer" OR "paroxetine hydrochloride hemihydrate" OR "paroxetine hydrochloride, hemihydrate" OR "paroxetine hydrochloride" OR paxpar):ti,ab,kw——149

#3 #1 OR #2——1036

#4 "panic disorder" [Mesh]——889

#5 （"panic attack" OR "disorder, panic" OR "disorders, panic" OR "panic disorders" OR "panic attacks" OR "attack, panic" OR "attacks, panic" OR "acute anxiety" OR panic OR fear）:ti,ab,kw——12966

#6 "agoraphobia" [Mesh]——411

#7 "agoraphobias":ti,ab,kw——964

#8 #4 OR #5 OR #6 OR #7——13102

#9 #3 AND #8——99

Search strategy in Scopus

#1 TITLE-ABS-KEY (paroxetine OR seroxat OR brisdelle OR FG-7051 OR LDMP OR pexeva OR paxil OR aropax OR 61869-08-7 OR BRL-29060 OR BRL29060 OR "BRL 29060 " OR "FG 7051" OR FG7051 OR "paroxetine acetate" OR "paroxetine hydrochloride anhydrous" OR "paroxetine maleate" OR "paroxetine, cis-(+)-Isomer" OR "paroxetine, cis-(-)-Isomer" OR "paroxetine, trans-(+)-Isomer" OR "paroxetine hydrochloride hemihydrate" OR "paroxetine hydrochloride, hemihydrate" OR "paroxetine hydrochloride" OR paxpar) AND ( LIMIT-TO ( LANGUAGE , "English" ) OR LIMIT-TO ( LANGUAGE , "Chinese" )——23643

#2 TITLE-ABS-KEY ("panic disorder" OR "panic attack" OR "disorder, panic" OR "disorders, panic" OR "panic disorders" OR "panic attacks" OR "attack, panic" OR "attacks, panic" OR "agoraphobia") AND ( LIMIT-TO (LANGUAGE , "English" ) OR LIMIT-TO ( LANGUAGE , "Chinese" )——16550

#3 TITLE-ABS-KEY ("randomized controlled trial" OR "controlled clinical trial" OR "controlled trial, randomized" OR "randomised controlled trial" OR "randomized controlled study" OR "controlled study" OR "multicenter study") AND (LIMIT-TO (LANGUAGE, "English") OR LIMIT-TO (LANGUAGE, "Chinese")——6147952

#4 #1 AND #2 AND #3——325

Search strategy in ScienceDirect

#1 (paroxetine OR seroxat OR FG-7051 OR LDMP OR paxil OR "paroxetine hydrochloride") AND ("panic disorder" OR "panic attack" OR "disorder, panic" OR "attack, panic" OR panic OR fear OR agoraphobia)——85

Search strategy in PsycINFO

#1 TI-AB(paroxetine OR seroxat OR cylert OR FG-7051 OR brisdelle OR LDMP OR pexeva OR paxil OR aropax OR BRL-29060 OR BRL29060 OR "BRL 29060" OR "FG 7051" OR FG7051 OR "paroxetine acetate" OR "paroxetine hydrochloride anhydrous" OR "paroxetine maleate" OR "paroxetine, cis-(+)-Isomer" OR "paroxetine, cis-(-)-Isomer" OR "paroxetine, trans-(+)-Isomer" OR "paroxetine hydrochloride hemihydrate" OR "paroxetine hydrochloride, hemihydrate" OR "paroxetine hydrochloride" OR paxpar) AND (language , "English" OR "Chinese" ) AND adulthood (18 yrs & older) AND human——1788

#2 (SU ( "panic disorder" OR "panic attack" OR "disorder, panic" OR "disorders, panic" OR "attack, panic" OR "attacks, panic" OR "panic disorders" OR "panic attacks" OR agoraphobia OR "acute anxiety" OR panic OR fear) AND (language , "English" OR "Chinese" ) AND adulthood (18 yrs & older) AND human——18603

#3 #1 AND #2——139

Search strategy in ClinicalTrials.gov

(paroxetine OR seroxat OR FG-7051 OR brisdelle OR LDMP OR pexeva OR paxil OR aropax OR BRL-29060 OR BRL29060 OR "BRL 29060" OR "FG 7051" OR FG7051 OR "paroxetine acetate" OR "paroxetine maleate" OR "paroxetine hydrochloride") AND ("panic attack" OR "disorder, panic" OR "disorders, panic" OR "panic disorders" OR "panic attacks" OR "attack, panic" OR "attacks, panic" OR "acute anxiety" OR agoraphobia OR panic OR fear OR "panic disorder") AND (interventional studies(clinical trials)) AND (adult and older adult (18 yrs & older))——14

Search strategy in Wanfang database

(Topic: (panic OR agoraphobia OR "phobic disorders" OR "panic disease" OR "panic disorder" OR "acute anxiety" OR "panic attack") AND (Topic:(paroxetine OR leyou OR shutanluo OR seroxat OR paxil OR cylert))——141

Search strategy in CNKI

(Topic: (panic OR agoraphobia OR "phobic disorders" OR "panic disease" OR "panic disorder" OR "acute anxiety" OR "panic attack") AND (Topic:(paroxetine OR leyou OR shutanluo OR seroxat OR paxil OR cylert))——66

Search strategy in Chongqing VIP

((panic OR agoraphobia OR "phobic disorders" OR "panic disease" OR "panic disorder" OR "acute anxiety" OR "panic attack") AND (paroxetine OR leyou OR shutanluo OR seroxat OR paxil OR cylert)):ti,kw——59

Search strategy in CBMdisc

(Common field: (panic OR agoraphobia OR "phobic disorders" OR "panic disease" OR "panic disorder" OR "acute anxiety")) AND (Common field: (paroxetine OR leyou OR shutanluo OR seroxat OR paxil OR cylert)) ——102

|  |
| --- |
