# Supplementary material for: Short-Term Efficacy and Tolerability of Paroxetine Versus Placebo for Panic Disorder: A Meta-Analysis of Randomized Controlled Trials
Source: Front Pharmacol. 2020 Mar 31;11:275. doi: 10.3389/fphar.2020.00275 (PMC7136560; doi:10.3389/fphar.2020.00275)
Supplement: Supplementary file 2 [file DataSheet_2.docx]

**Supplementary S2 file.** Sensitivity analyses

zero full panic attacks

avoidance

CGI-I

CGI-S
